# Association between body mass index and physical fitness index among middle and high school students aged 13–18 in Shanxi Province: a cross-sectional study

**DOI:** 10.3389/fpubh.2026.1880008

**Published:** 2026-07-21

**Authors:** Shaochen Zhou, Dingyu Zhao, Hao Luo

**Affiliations:** Department of Physical Education, Shanxi University, Taiyuan, Shanxi, China

**Keywords:** adolescent, BMI-body mass index, inverted U-shaped relationship, PFI, school health

## Abstract

**Objective:**

To investigate the relationship between Body Mass Index (BMI) and Physical Fitness Index (PFI) among middle and high school students aged 13–18 in Shanxi Province, and to provide a reference for adolescent physical health promotion.

**Methods:**

A total of 3,600 middle and high school students aged 13–18 (1,800 boys and 1,800 girls) from Shanxi Province who participated in the 2025 National Physical Fitness and Health Survey were selected as the study subjects. They were divided into underweight, normal weight, overweight, and obese groups based on the Chinese screening standards for overweight, obesity, and malnutrition in school-age children and adolescents. The Z-score method was used to calculate the Z-scores for each physical fitness indicator, which were then summed to obtain the Physical Fitness Index (PFI), comprehensively reflecting physical fitness status. Differences in physical fitness indicators among students of different genders and different BMI groups were compared, and the association between BMI and PFI was examined using a quadratic polynomial regression model, in which age was included as a covariate and BMI was centered to minimize multicollinearity.

**Results:**

Analysis of data from middle and high school students aged 13–18 revealed significant differences in PFI among different BMI groups (*P* < 0.05). The normal weight group had the highest PFI (boys: 0.37 ± 3.61, girls: 0.15 ± 3.33), while the underweight and obese groups had significantly lower PFI. BMI and PFI showed an inverted “U”-shaped distribution. Furthermore, except for vital capacity and grip strength, most physical fitness indicators were optimal in the normal weight group. Overweight and obese groups performed poorly in endurance running and standing long jump, among other events. With increasing age, BMI and most physical fitness indicators significantly improved (*P* < 0.01). Gender comparison showed boys had advantages in strength and explosive power events, while girls had better flexibility, with significant differences (*P* < 0.01). Regression analysis showed that, after controlling for age, the regression coefficient of BMI_C^2^ was negative (boys: *B* = −0.036, *P* < 0.01; girls: *B* = −0.026, *P* < 0.01), indicating that PFI is a quadratic function of BMI, with PFI first increasing and then decreasing as BMI increases.

**Conclusion:**

Based on the 2025 physical fitness survey data from Shanxi Province, this study found an inverted U-shaped association between BMI and PFI among middle and high school students aged 13–18, with the normal weight range corresponding to the peak PFI, while both underweight and overweight/obesity were associated with lower PFI. This association remained significant after controlling for age, but had limited explanatory power in girls. It is recommended that school physical education programs address both underweight and overweight/obese students with targeted interventions, and consider gender differences when developing intervention strategies. Future studies should include variables such as physical activity, dietary habits, and body fat percentage, and adopt longitudinal designs to verify the directionality of the association.

## 1 Introduction

The physical fitness and health of children and adolescents are not only the foundation of national health but also bear upon the future of the nation and the long-term development of society. However, the current state of Chinese adolescents' physical health is concerning, marked by the dual challenges of soaring myopia and obesity rates, coupled with declining physical fitness and cardiovascular function (1). This not only affects the health status and quality of life of individual adolescents, but also poses a serious challenge to the implementation of the “Healthy China” strategy (2). Since the initiation of the National Student Physical Fitness and Health Survey in 1985, the overall physical fitness status of Chinese adolescents has shown a fluctuating downward trend, with most physical fitness indicators continuously declining before 2005. Starting in 2010, the decline stopped and began to rebound, showing an upward inflection point (3). Nevertheless, the excellent rate of student physical health standards in most regions of the country remains significantly low, still showing a certain gap from the target requirements of the “Healthy China 2030” planning outline (4). As representatives of the broader adolescent population, the development of physical fitness levels in middle school students has always received widespread social attention. Factors such as increased academic pressure, increased sedentary behavior, and insufficient exercise time may exacerbate the dual risks of declining physical fitness and abnormal weight, making it particularly important to focus on the association between their body morphology and physical fitness performance (5).

Body Mass Index (BMI), as a commonly used indicator reflecting nutritional status and body composition, has been extensively studied in relation to physical fitness, which has become a key topic in physical health research. Previous studies have shown that the relationship between BMI and physical fitness is not simply linear, but rather exhibits an “optimal range,” with both low and high BMI values being associated with lower fitness levels. However, most previous studies failed to adjust for age as an important confounding variable in regression models, nor did they adequately address the multicollinearity between BMI and BMI^2^. Moreover, the optimal BMI ranges identified across different regions and age groups have been inconsistent, warranting further validation using data from more diverse populations. To address these gaps, the present study, based on data from the 2025 National Student Physical Fitness and Health Survey in Shanxi Province, employed a quadratic polynomial regression model to examine the association between BMI and PFI among middle and high school students aged 13–18, while controlling for age and centering BMI to reduce multicollinearity. We also explored the effects of different BMI categories on various physical fitness indicators, aiming to provide empirical evidence for adolescent physical health promotion.

## 2 Data sources and methods

### 2.1 Data sources

Data were sourced from the Shanxi Province 2025 National Student Physical Fitness and Health Survey conducted in October-November 2025. According to the requirements of the “Notice from the General Office of the Ministry of Education and Four Other Departments on Conducting the Ninth National Student Physical Fitness and Health Survey,” a stratified random cluster sampling method was used. Survey schools were first determined, then stratified by grade, and random cluster sampling was conducted with teaching classes as the unit to form the survey sample. In each survey, the ratio of gender and urban/rural primary and secondary school students was approximately 1:1. The number of participants from three economic regions—good (Taiyuan City), medium (Datong City), and poor (Yuncheng City)—was approximately 1:1:1. Students with severe organic diseases, developmental abnormalities, disabilities, or acute illnesses were excluded. This paper selected relevant data from middle school students aged 13–18 (1,800 boys, 1,800 girls) for analysis.

### 2.2 Methods

#### 2.2.1 Physical fitness indicator measurement

Strictly following the requirements of the “National Student Physical Health Standard (2014 Revision)” and the survey manual, trained testers conducted physical examinations and physical fitness tests on students. Main indicators included: height, weight, vital capacity, grip strength, sit-and-reach, standing long jump, sit-ups (girls), pull-ups (boys), 50-m run, 800-m run (girls), and 1,000-m run (boys).

#### 2.2.2 Health status determination

Based on the “Screening for Malnutrition in School-age Children and Adolescents (WS/T 456-−2014)” (6) and “Screening for Overweight and Obesity in School-age Children and Adolescents (WS/T 586-−2018) (7)”, Body Mass Index (BMI) was calculated and categorized into underweight, normal weight, overweight, and obese groups.

#### 2.2.3 Physical Fitness Index (PFI) calculation

The Z-score method was used to standardize each physical fitness indicator. The calculation formula was: PFI = Z_vital capacity + Z_grip strength + Z_standing long jump + Z_sit-and-reach – Z_50 m run – Z_endurance run. For running events, because shorter times indicate better performance, the opposite of their Z-score was used in the calculation (8). A positive and larger PFI value indicates better comprehensive physical fitness.

#### 2.2.4 Statistical methods

All data were entered using the “National Student Physical Health Survey Data Entry and Statistical System.” SPSS 27.0 software was used for statistical analysis. Before analysis, logical consistency checks were performed on the data to identify and handle possible abnormal values or entry errors, ensuring data quality. Measurement data are expressed as mean ± standard deviation (x ± s).

Independent-sample *t*-tests were used to compare the differences in physical fitness indicators between genders. One-way analysis of variance (ANOVA) was employed to compare the differences in physical fitness indicators across BMI groups. When the ANOVA results were statistically significant, pairwise *post-hoc* comparisons were conducted using the Tukey method.

To explore the quantitative relationship between BMI and PFI, a quadratic polynomial regression model was constructed in this study. To reduce the multicollinearity between BMI and its squared term (BMI^2^), BMI was centered (BMI_C = BMI – overall mean BMI), and a centered squared term (BMI_C^2^ = BMI_C × BMI_C) was then created. PFI was set as the dependent variable, and BMI_C, BMI_C^2^, and age were entered as independent variables, yielding the regression model: PFI = a × BMI_C^2^ + b × BMI_C + c × age + d. The model reports unstandardized regression coefficients (B), standard errors (SE), 95% confidence intervals (CI), variance inflation factors (VIF), and the coefficient of determination (*R*^2^). All statistical analyses used *P* < 0.05 as the standard for statistical significance.

## 3 Results

### 3.1 Comparison of physical fitness indicators between genders

A total of 3,600 participants were included in this study, consisting of 1,800 males and 1,800 females, with a balanced gender ratio. The comparisons of BMI and physical fitness indicators between male and female students are presented in Table 1.

**Table 1:** Comparison of physical fitness indicators between male and female students (Mean ± SD).

Variable	Male students (*n* = 1,800)	Female students (*n* = 1,800)	*t*	*P*
Age (years)	15.5 ± 1.8	15.5 ± 1.7	–	–
BMI (kg/m^2^)	21.39 ± 3.72	21.03 ± 3.13	3.14	< 0.01
Vital capacity (mL)	4013.08 ± 958.74	2868.44 ± 662.20	41.68	< 0.01
Grip strength (N)	36.38 ± 8.85	23.33 ± 4.88	54.79	< 0.01
Sit-and-reach (cm)	10.42 ± 7.75	14.01 ± 7.64	−14.01	< 0.01
Standing long jump (cm)	216.50 ± 29.45	160.74 ± 21.55	64.83	< 0.01
50 m run (s)	7.84 ± 0.89	9.70 ± 2.35	−31.43	< 0.01
Endurance run (s)^*^	260.60 ± 43.03	258.57 ± 36.41	1.53	0.13
PFI	0.00 ± 3.84	0.00 ± 3.29	0	1

^*^Male students performed the 1,000-m run, while female students performed the 800-m run. Age was a matching variable and was not tested for between-group differences.

The results showed that the mean BMI of male students was significantly higher than that of female students (*t* = 3.14, *P* < 0.01). In terms of physical fitness indicators, male students performed significantly better in vital capacity, grip strength, standing long jump, and the 50-m run (all *P* < 0.01); female students performed significantly better in the sit-and-reach test (*t* = −14.01, *P* < 0.01). No statistically significant differences were observed between males and females in the endurance run (1,000 m for males/800 m for females) or the comprehensive Physical Fitness Index (PFI) (*P* > 0.05).

### 3.2 Comparison of PFI and key physical fitness indicators across BMI groups

According to the Chinese screening criteria for overweight, obesity, and malnutrition in school-age children and adolescents, participants were divided into four groups: underweight, normal weight, overweight, and obesity. The comparisons of PFI and key physical fitness indicators across BMI groups are presented in Table 2.

**Table 2:** Comparison of physical fitness indicators across BMI groups (Mean ± SD).

Gender	BMI Group	*N*	Statistics	Vital capacity (mL)	Grip strength (kg)	50 m Run (s)	PFI
**Male students**	Underweight	102		3,363.97 ± 797.08	30.74 ± 8.05	264.48 ± 44.60	−1.95 ± 4.32
	Normal	1,234		4,010.99 ± 912.76^*^	35.73 ± 8.41^*^	252.66 ± 35.61^*^	0.37 ± 3.61^*^
	Overweight	317		4,107.95 ± 1,040.06^*^	38.79 ± 9.01#	268.45 ± 42.45#	0.03 ± 3.80
	Obese	147		4,276.50 ± 1,060.90#	40.58 ± 9.57##	307.56 ± 62.73##	−1.77 ± 4.49#&
			F	21.00	36.94	87.07	23.86
			P	< 0.01	< 0.01	< 0.01	< 0.01
**Female students**	Underweight	57		2,517.60 ± 629.44	20.12 ± 4.68	255.14 ± 35.60	−1.43 ± 2.97
	Normal	1377		2,852.28 ± 645.99^*^	23.03 ± 4.69^*^	254.71 ± 35.61	0.15 ± 3.33^*^
	Overweight	277		2,958.24 ± 681.87^*^	24.61 ± 4.96##	267.20 ± 33.30##	−0.06 ± 3.01^*^
	Obese	89		3,063.73 ± 762.42##	25.88 ± 5.70##	293.52 ± 35.87##&	−1.18 ± 3.27#&
			F	10.03	25.41	40.32	8.41
			P	< 0.01	< 0.01	< 0.01	< 0.01

^*^Male students performed the 1000-m run, while female students performed the 800-meter run. *P* < 0.05 compared with the underweight group of the same sex; #*P* < 0.05 compared with the normal-weight group of the same sex; &*P* < 0.05 compared with the overweight group of the same sex (Tukey's *post-hoc* test).

The results showed that PFI differed significantly across BMI groups in both male and female students (all *P* < 0.01). The normal-weight group had the highest PFI (males: 0.37 ± 3.61; females: 0.15 ± 3.33), while both the underweight and obesity groups had significantly lower PFI than the normal-weight group. The PFI values for the underweight, normal-weight, overweight, and obesity groups were −1.95, 0.37, 0.03, and –1.77 in males, and −1.43, 0.15, −0.06, and −1.18 in females, respectively, showing an inverted U-shaped trend in which PFI first increased and then decreased with increasing BMI.

Of note, vital capacity and grip strength showed a continuous upward trend with increasing BMI, with the obesity group scoring significantly higher than both the normal-weight and underweight groups (*P* < 0.05). In contrast, endurance run performance was best in the normal-weight group, while the obesity group performed significantly worse than the normal-weight group (*P* < 0.05). This pattern suggests a dissociation between absolute strength-related indicators and relative aerobic endurance in the obesity group.

### 3.3 Quadratic polynomial regression analysis of BMI and PFI

To control for the confounding effect of age and to verify the non-linear relationship between BMI and PFI, a quadratic polynomial regression model was constructed with PFI as the dependent variable and centered BMI (BMI_C), the squared term of centered BMI (BMI_C^2^), and age as independent variables. The results are presented in Table 3.

**Table 3:** Results of quadratic polynomial regression analysis for BMI and PFI (with age adjusted).

Gender	Variable	Unstandardized		Standardized	*t*	*p*	95% Confidence interval for B				
		*B*	SE	Beta			Lower bound	Upper bound	Tolerance	VIF	Model *R*^2^
**Male students**	(Constant)	−15.821	0.737		−21.465	0.000	−17.267	−14.376			0.281
	BMI_C	0.102	0.027	0.099	3.819	0.000	0.049	0.154	0.601	1.663	
	$BMI_C^2^$	−0.036	0.003	−0.258	−10.315	0.000	−0.042	−0.029	0.640	1.563	
	Age	1.052	0.047	0.469	22.509	0.000	0.961	1.144	0.920	1.087	
**Female students**	(Constant)	−6.065	0.707		−8.575	0.000	−7.452	−4.678			0.069
	BMI_C	0.080	0.028	0.076	2.842	0.005	0.025	0.136	0.717	1.394	
	$BMI_C^2^$	−0.026	0.004	−0.154	−5.840	0.000	−0.034	−0.017	0.745	1.342	
	age	0.407	0.045	0.211	9.055	0.000	0.319	0.496	0.951	1.051	

BMI_C, centered BMI (BMI minus mean BMI); BMI_C^2^, squared term of centered BMI; VIF, Variance Inflation Factor. Adjusted *R*^2^ for males = 0.280; for females = 0.068.

The results showed that the regression coefficients for both BMI_C and BMI_C^2^ were statistically significant in both males and females (all *P* < 0.01), and the quadratic term coefficients were negative (male: −0.036; female: −0.026), confirming an inverted U-shaped relationship between PFI and BMI.

The VIF values for all independent variables ranged from 1.01 to 1.66, well below the threshold of 10, indicating that multicollinearity was not a concern in the model.

The coefficient of determination (*R*^2^) for the male model was 0.281 (adjusted *R*^2^ = 0.280), while for the female model it was 0.069 (adjusted *R*^2^ = 0.068), suggesting that BMI and age together explained approximately 28% of the variance in PFI in males, but had relatively limited explanatory power in females.

Based on the regression equations, the estimated BMI corresponding to the peak PFI was approximately 22.8 kg/m^2^ for males and 22.6 kg/m^2^ for females.

The regression equations for males and females were as follows:


$$\begin{array}{c} \text{M}\text{a}\text{l}\text{e}\text{s}:\text{P}\text{F}\text{I}=-0.036\times BMI\text{\_}C^{2}+0.102\times BMI\text{\_}C \end{array}$$

$$\begin{array}{c} +1.052\times Age\;-15.821 \end{array}$$ (1)

$$\begin{array}{c} \text{F}\text{e}\text{m}\text{a}\text{l}\text{e}\text{s}:\text{P}\text{F}\text{I}=-0.026\times BMI\text{\_}C^{2}+0.080\times BMI\text{\_}C \end{array}$$

$$\begin{array}{c} +0.407\times Age\;-6.065 \end{array}$$ (2)

## 4 Discussion

This study, based on the 2025 physical fitness and health survey data of middle and high school students aged 13–18 in Shanxi Province, systematically analyzed the associations between different BMI categories and PFI as well as various physical fitness indicators. Three core findings emerged: First, there was an inverted U-shaped relationship between BMI and PFI, with the normal-weight group showing the highest PFI; second, the obesity group unexpectedly outperformed the normal-weight group in absolute strength indicators such as vital capacity and grip strength, but performed the worst in the endurance run; third, BMI and age together explained substantially more variance in PFI for males (*R*^2^ = 28.1%) than for females (*R*^2^ = 6.9%). Before interpreting these results, it is important to clarify that this study employed a cross-sectional design, and all observed associations do not imply causal relationships between BMI and physical fitness. For instance, the observed association between low BMI and low PFI could be attributed to low body weight leading to poor fitness, or conversely, poor fitness (e.g., low muscle mass) leading to low body weight, or even confounding by third variables (e.g., chronic illness, inadequate nutritional intake). Therefore, all subsequent discussion is limited to the level of association and should not be construed as causal inference.

### 4.1 Inverted U-shaped relationship between BMI and PFI and the optimal BMI range

The present study found that PFI exhibited an inverted U-shaped trend, first increasing and then decreasing with increasing BMI, and this pattern was highly consistent across both male and female students. The normal-weight group had the highest PFI (males: 0.37 ± 3.61; females: 0.15 ± 3.33), while both the underweight group (males: −1.95 ± 4.32; females: −1.43 ± 2.97) and the obesity group (males: −1.77 ± 4.49; females: −1.18 ± 3.27) had significantly lower PFI than the normal-weight group. The quadratic polynomial regression model further confirmed this non-linear relationship, with negative coefficients for BMI_C^2^ after adjusting for age (males: −0.036; females: −0.026; all *P* < 0.01), confirming that PFI is a quadratic function of BMI. Based on the regression equations, the BMI values corresponding to the peak PFI were estimated to be approximately 22.8 kg/m^2^ for males and 22.6 kg/m^2^ for females, both falling near the upper limit of the normal weight range.

This finding is consistent with previous studies. A study on students aged 7–18 in Anhui Province similarly found that the normal BMI group had the highest PFI, while both low and high BMI were associated with decreased physical fitness, showing a typical parabolic trend (9). Another study on students aged 7–18 in Henan Province also pointed out that BMI and physical fitness showed a quadratic function relationship opening downward, with a stable inverted “U”-shaped distribution across different school stages (10).The peak BMI values observed in this study (22.8 kg/m^2^ for males and 22.6 kg/m^2^ for females) were slightly higher than those reported in previous studies (approximately 19–20 kg/m^2^). Three possible reasons may account for this discrepancy. First, the age composition differs—our study focused on adolescents aged 13–18 years, who are in the late pubertal stage and have significantly higher muscle mass and bone mineral density than childhood samples, which naturally shifts the optimal BMI range upward. Second, regional and economic differences may play a role: our sample was drawn from Shanxi Province, whereas most previous studies were conducted in eastern or central China, where dietary patterns and physical activity levels may differ, potentially leading to variations in baseline body composition. Third, the statistical approaches vary—our study included age as a covariate in the regression model, whereas some previous studies did not control for age confounding. Collectively, these comparisons suggest that the inverted U-shaped BMI–PFI relationship is relatively stable across samples; however, extrapolation of the specific optimal BMI range to other populations should take into account differences in age, region, and body composition.

The inverted U-shaped relationship between BMI and PFI may be related to the bidirectional effects of body composition on physical performance.maintaining BMI within the normal range usually means having a reasonable ratio of muscle mass to fat, which helps the coordinated development of strength, explosive power, and cardiorespiratory endurance, ensuring optimal physical fitness levels (11). Although overweight and obese individuals may perform better in indicators like vital capacity and grip strength, excessive body fat increases exercise load, reduces cardiovascular endurance, muscle strength, muscle endurance, and explosive power (12), mainly manifested in poorer performance in endurance running, pull-ups, sit-and-reach, 50 m run, and standing long jump. Furthermore, studies have shown that underweight individuals have insufficient muscle mass, leading to poor muscle explosive power and abdominal muscle endurance, reflected in poor standing long jump performance (13), This was further corroborated by the finding that the underweight group had lower standing long jump performance than the normal-weight group in both males (217.03 ± 35.05 cm vs. 220.50 ± 28.36 cm) and females (159.77 ± 22.96 cm vs. 161.49 ± 21.42 cm).

### 4.2 The paradox of “strength advantage” and “endurance disadvantage” in the obesity group

A noteworthy paradox emerged in this study: the obesity group had significantly higher vital capacity (males: 4,276.50 ± 1,060.90 mL; females: 3,063.73 ± 762.42 mL) and grip strength (males: 40.58 ± 9.57 kg; females: 25.88 ± 5.70 kg) than the normal-weight group (vital capacity: males: 4,010.99 ± 912.76 mL; females: 2,852.28 ± 645.99 mL; grip strength: males: 35.73 ± 8.41 kg; females: 23.03 ± 4.69 kg), but performed significantly worse in the endurance run (males: 307.56 ± 62.73 s vs. 252.66 ± 35.61 s; females: 293.52 ± 35.87 s vs. 254.71 ± 35.61 s). This divergent pattern suggests that vital capacity and grip strength reflect physiological functions related to absolute body size rather than relative physical fitness levels. The greater body mass in the obesity group inherently requires higher pulmonary ventilation and absolute strength to support daily activities; however, cardiorespiratory endurance and exercise efficiency (e.g., oxygen uptake per unit body weight) did not improve correspondingly. This finding also implies that using absolute indicators alone to evaluate physical fitness may mask the true disadvantages of obese individuals in relative motor capacity, and that multidimensional composite indices such as PFI are needed to more comprehensively reflect the actual physical fitness level of adolescents.

Furthermore, the inherent limitations of BMI as an indirect indicator of body composition should be acknowledged. BMI cannot differentiate between fat mass and muscle mass; a student with low body fat percentage but high muscle mass may be classified as “overweight” due to a relatively high BMI, yet their physical fitness may be superior to that of peers with normal weight but high body fat percentage. This may partially explain why the overweight group in this study did not exhibit the poorest fitness, and why the obesity group paradoxically outperformed others in absolute strength measures. Therefore, the validity of BMI as a predictor of physical fitness may be overestimated in populations with substantial variation in body composition. Future studies are encouraged to incorporate indicators such as waist circumference and body fat percentage to more precisely delineate the relationship between body composition and physical fitness.

### 4.3 Gender and age differences

The results of this study further confirmed that there are significant differences in physical fitness profiles between male and female students. Males performed significantly better than females in strength- and speed-dominated indicators such as vital capacity, grip strength, standing long jump, and the 50-m run, while females showed a significant advantage in flexibility-related indicators such as the sit-and-reach test (all *P* < 0.01). This gender difference is consistent with the physiological effects of pubertal hormones: androgens promote muscle development, whereas estrogens influence fat deposition and joint flexibility (14, 15).In formulating future intervention strategies for adolescent physical fitness, full consideration should be given to gender differences: female students may appropriately incorporate strength training, while male students should enhance flexibility and coordination exercises. Regarding age-related trends, BMI and most physical fitness indicators showed an upward trajectory with increasing age, which is consistent with previous findings (16). However, in the present study, male students' performance in the 50-m run and 1,000-m run plateaued or even slightly declined between the ages of 17 and 18, suggesting that during the stage of increasing academic pressure in senior high school, greater attention should be paid to the continuity and variety of physical exercise to prevent a “disconnect” in fitness development.

### 4.4 Limitations

Several limitations of this study should be considered when interpreting the findings.

First, the cross-sectional design can only reveal associations between BMI and PFI, and cannot establish causal relationships. For instance, low BMI may lead to poor physical fitness, but poor fitness (e.g., low muscle mass) could also result in low BMI, and the two may have a bidirectional relationship. Future studies employing longitudinal tracking or intervention designs are needed to validate the directionality of these associations.

Second, the sample was drawn exclusively from Shanxi Province, so the generalizability of the findings requires caution. Differences in lifestyle, dietary habits, and physical education resources across regions may influence the specific pattern of the BMI–fitness relationship. Multi-center and cross-regional studies are recommended to verify the universality of our findings.

Third, the low *R*^2^ value for females (0.069) in the regression model suggests that BMI and age have limited explanatory power for PFI in females. This may be attributable to the greater biological complexity of female physical fitness determinants (e.g., menstrual cycle, body fat distribution patterns), or may indicate that other unmeasured variables exert a stronger influence on fitness in females. Future research should incorporate a more comprehensive set of variables.

Fourth, this study did not include key confounding variables such as physical activity level, dietary patterns, sleep quality, pubertal stage, or socioeconomic status—factors that may affect both BMI and physical fitness independently. The absence of these variables may have introduced confounding bias.

Fifth, the PFI was constructed as an equally weighted sum of Z-scores for each indicator, which implicitly assumes that all indicators are equally important and does not account for correlations among indicators or the biasing effects of body weight on vital capacity and grip strength. Furthermore, BMI, as an indirect measure of body composition, cannot distinguish between fat mass and muscle mass; a student with high muscle mass but low body fat percentage may be misclassified as “overweight.” Future studies could explore alternative approaches such as weighted Z-scores or principal component analysis, and incorporate more precise body composition measures such as waist circumference and body fat percentage.

## Data Availability

The original contributions presented in the study are included in the article/Supplementary material, further inquiries can be directed to the corresponding author.

## References

[1] Ji L Yin XJ Wu HP Yang XF Liu Y. An exploratory study on the evaluation standards of physical health for children and adolescents in China under the background of “integration of sports and education”. Sport Sci. (2021) 41:42–54. doi: 10.16469/j.css.202103006

[2] Shi CY Dou L Guo LL Shen HJ. Investigation and influencing factors of physical health knowledge level of high school students in Jiangsu Province. Sport Sci. (2023) 44:84–92. doi: 10.13598/j.issn1004-4590.2023.05.006

[3] Zhang Y He L. Dynamic analysis of physical health status of Chinese adolescents—based on four national physical health monitoring data from 2000 to 2014. China Youth Study. (2016) 5–12. doi: 10.19633/j.cnki.11-2579/d.2016.06.001

[4] Song Y Luo DM Hu PJ Yan XJ Zhang JS Lei YT . Trend analysis of excellent rate of physical health standards for Han Chinese middle school students aged 13–18 from 1985 to 2014. J Peking Univ. (2020) 52:317–22. doi: 10.19723/j.issn.1671-167x.2020.02.020 PMC7433453 32306017

[5] Zhen ZP Zhu WM Yao MY. Current status and trends of international research on children and adolescents' physical fitness and health promotion—inspiration from the 63rd annual meeting of the American college of sports medicine. J Beijing Sport Univ. (2016) 39:44–50. doi: 10.19582/j.cnki.11-3785/g8.2016.08.008

[6] National Health and Family Planning Commission. Screening for Malnutrition in School-Age Children and Adolescents: WS/T456-2014. Beijing: China Standards Press (2014).

[7] National Health and Family Planning Commission. Screening Standards for Overweight and Obesity in School-Age Children and Adolescents: WS/T586-2018. Beijing: China Standards Press (2018).

[8] Huang YC Malina RM. Body mass index (BMI) and health-related physical fitness in Taiwanese youth 9-18 years. Med Sci Sports Exerc. (2007) 39:701–8. doi: 10.1249/mss.0b013e31802f0512 17414809

[9] Zhao YQ Wang FY Zhu P. Study on the correlation between body mass index (BMI) and physical fitness index in children and adolescents. Chin J Epidemiol. (2012) 33:265–8. 22613375

[10] Yang ML Sun J Yan GL Luo XM Peng YL. Relationship between body mass index (BMI) and physical fitness in students aged 7–18. Chin J Sch Health. (2018) 39:688–93. doi: 10.16835/j.cnki.1000-9817.2018.05.014

[11] Shao J Sun Y Yin XJ Li YQ Suzuki A. Relationship between body mass index (BMI) and physical fitness index in children and adolescents in China and Japan. Chin J Sch Health. (2019) 40:1616–9. doi: 10.16835/j.cnki.1000-9817.2019.11.005

[12] Casonatto J Fernandes AR Batista BM Cyrino ES Coelho-E-Silva MJ de Arruda M . Association between health-related physical fitness and body mass index status in children. J Child Health Care. (2016) 20:294–303. doi: 10.1177/1367493515598645 26396021

[13] Evi V Dané C Bouwien ES. Underweight children are agile but lack power. BMC Pediatr. (2022) 22:490. doi: 10.1186/s12887-022-03544-3 35982448 PMC9386997

[14] Teng JL. Correlation analysis between different body mass index (BMI) and physical fitness indicators and physical fitness index in children and adolescents in Nanjing. Mod Prev Med. (2019) 46:3709–13.

[15] Li HJ Yang L Zhang N Zhuang C Kong ZX Zhang YM. Analysis of body fat percentage percentile reference values using the LMS method for children and adolescents aged 6–18 in Beijing. Chin J Sch Health. (2015) 36:815–7. doi: 10.16835/j.cnki.1000-9817.2015.06.007

[16] Fu BW An TY Lou XM . Physical fitness status and influencing factors of primary and secondary school students in Henan Province in 2019. Chin J Sch Health. (2023) 44:99–109. doi: 10.16835/j.cnki.1000-9817.2023.01.022

